# Supplementary material for: Obesity-Related Brain Cholinergic System Impairment in High-Fat-Diet-Fed Rats
Source: Nutrients. 2022 Mar 15;14(6):1243. doi: 10.3390/nu14061243 (PMC8948807; doi:10.3390/nu14061243)
Supplement: Supplementary file 1 [file nutrients-14-01243-s001.zip › nutrients-1594198-supplementary.pdf]

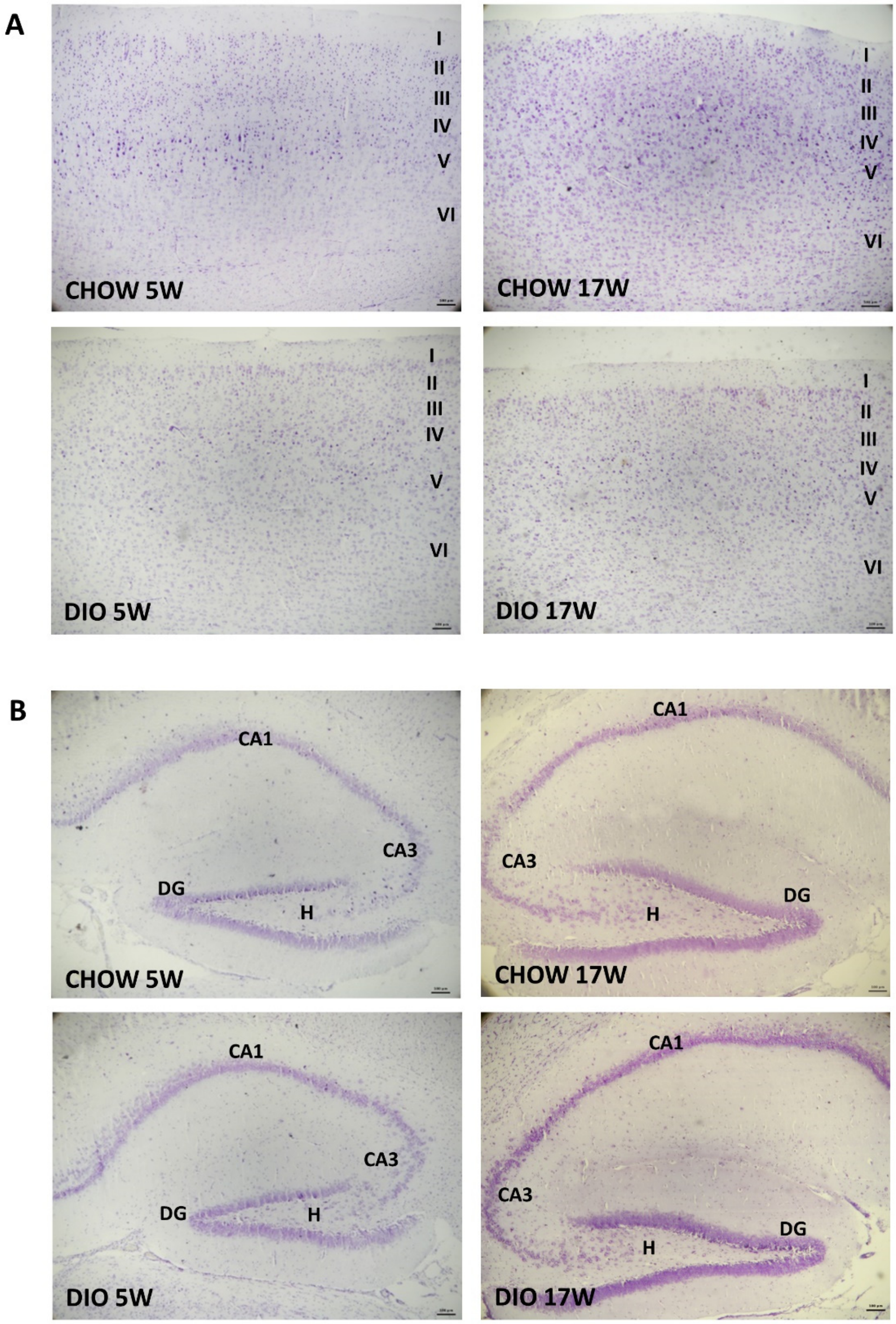

Supplementary Figure S1. Sections of rat frontal cortex (**A**) and hippocampus (**B**) stained with cresyl violet. Roman numerals indicate the layers of cerebral cortex. CA1: field CA1 of hippocampus: CA3: field CA3 of hippocampus; DG: dentate gyrus; H: hilus of the dentate gyrus. Scale bar: 100 μm.

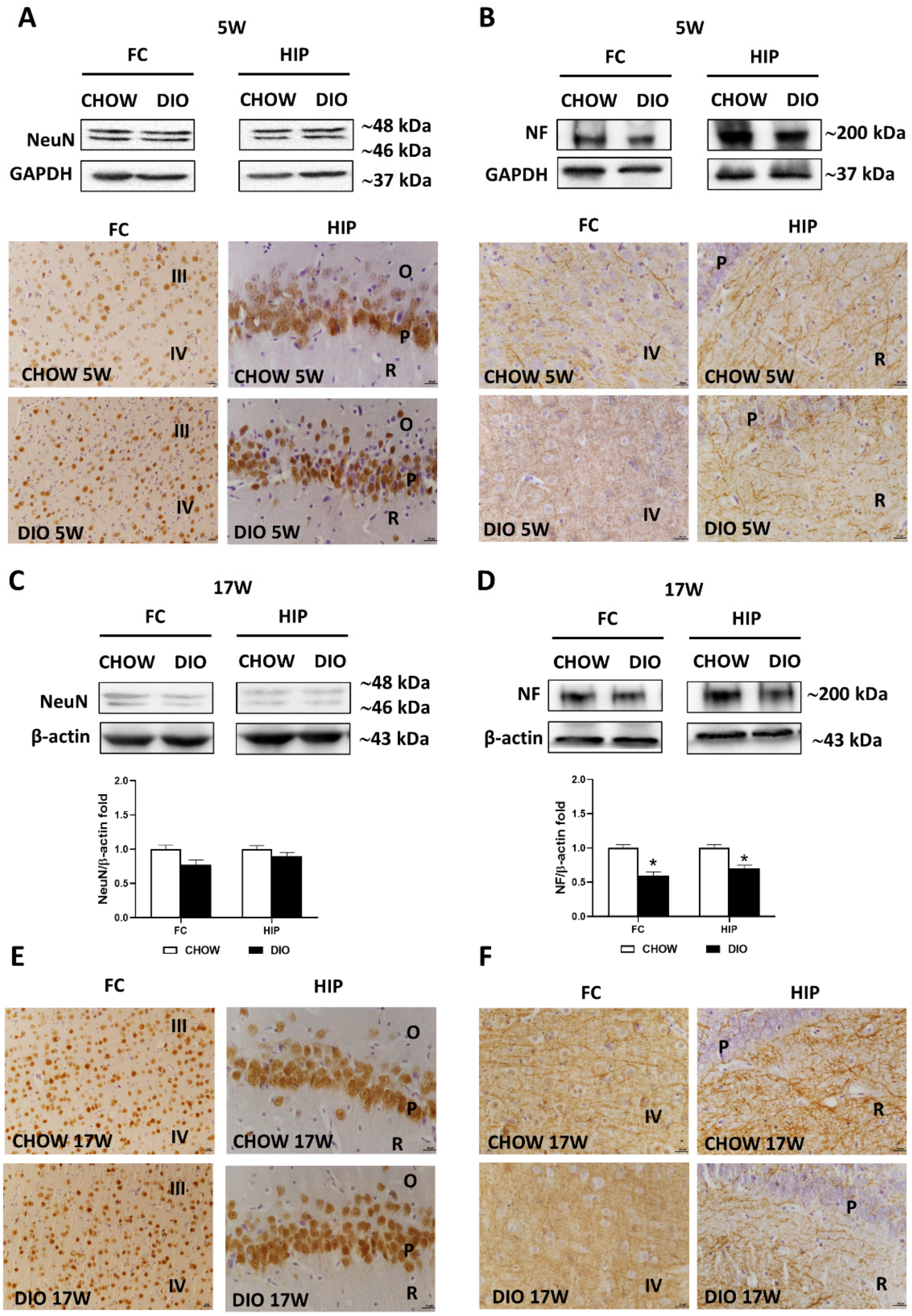

Supplementary Figure S2. Immunoblotting and immunoreaction of neuronal nuclei (NeuN) and neurofilament 200kDa (NF). Lysates and representative pictures in the frontal cortex (FC), and hippocampus (HIP) from rats fed with a standard diet (CHOW) and high-fat diet (DIO) for 5 weeks, were immunoblotted and stained using specific anti-NeuN (**A**) and anti-NF (**B**). Lysates and representative pictures in FC, and HIP from CHOW and DIO rats after 17 weeks were immunoblotted and stained using specific anti-NeuN (**C, E**) and anti-NF (**D, F**). Bar graphs indicate the densitometric analysis using CHOW as control, and β-actin was used as a reference loading protein. Data are mean ± S.E.M. * $p < 0.05$ *vs.* age-matched CHOW. III, IV: third and fourth layer of frontal cortex; O: *stratum oriens*; P: pyramidal cells; R: *stratum radiatum*. Scale bar: 25 μm.
